# Spinal segments do not move together predictably during daily activities

**DOI:** 10.1016/j.gaitpost.2018.10.031

**Published:** 2019-01

**Authors:** Enrica Papi, Anthony M.J. Bull, Alison H. McGregor

**Affiliations:** a Department of Surgery and Cancer, Imperial College London, London, UK; b Department of Bioengineering, Imperial College London, London, UK

**Keywords:** Cross-correlation, Low back pain, Motion analysis, Kinematics, Multi-segment

## Abstract

Background: Considering the thoracic, lumbar spine or whole spine as rigid segments has been the norm until recent studies highlighted the importance of more detailed modelling. A better understanding of the requirement for spine multi-segmental analysis could guide planning of future studies and avoid missing clinically-relevant information.

Research question: This study aims to assess the correlation between adjacent spine segments movement thereby evaluating segmental redundancy in both healthy and participants with low back pain (LBP).

Methods: A 3D motion capture system tracked the movement of upper and lower thoracic and lumbar spine segments in twenty healthy and twenty participants with LBP. Tasks performed included walking, sit-to-stand and lifting, repeated 3 times. 3D angular kinematics were calculated for each spine segment. Segmental redundancy was evaluated through cross-correlation (R_xy_) analysis of kinematics time series and correlation of range of motion (R_ROM_) of adjacent spine segments.

Results: The upper/lower lumbar pairing showed weak correlations in the LBP group for all tasks and anatomical planes (R_xy_range:0.02–0.36) but moderate and strong correlations during walking (R_xy_ _frontalplane:0.4) and lifting (R_xy_ _sagittalplane:0.64) in the healthy group. The lower thoracic/upper lumbar pairing had weak correlations for both groups during lifting and sit-to-stand in the frontal plane and for walking (R_xy_:0.01) in the sagittal plane only. The upper/lower thoracic pairing had moderate correlations during sit-to-stand in sagittal and transverse plane in patients with LBP (R_xy_ _sagittalplane:0.41; R_xy_ _transverse plane:−0.42) but weak in healthy (R_xy_ _sagittalplane:0.23; R_xy_ _transverseplane:−0.34); the contrary was observed during lifting.

The majority of R_ROM_ values (55/72) demonstrated weak correlations.

Significance:The results suggest that multi-segmental analysis of the spine is necessary if spine movement characteristics are to be fully understood. We cannot establish *a priori* where redundancy occurs based on healthy data, therefore extra consideration should be made when planning studies with pathological cohorts.

## Highlights

• We cannot predict spine kinematics segmental redundancy *a priori*.
• Tasks, anatomical planes and participant group affect spine segments correlations.
• Spine multi-segmental analysis is required to better understand spine movement.

## 1 Introduction

Movement analysis is widely performed to assess spine function in people affected by low back pain (LBP). The importance of spinal kinematic assessment in enhancing the understanding of the mechanical factors associated with LBP is widely recognised [1,2], yet there are contrasting results emerging from the literature when comparing LBP to healthy subjects [3]. A recent review highlighted one of the reasons to be the different modelling approaches adopted to assess spinal kinematics [3]. The majority of studies lack a multi-segmental approach with most viewing the lumbar spine as a rigid single segment. This goes against recent studies which suggest that, for some instances only, the upper and lower lumbar spine segments move differently, and that experimental consideration of this increases the ability of biomechanical studies to differentiate between motion patterns of LBP and healthy populations [[3], [4], [5], [6], [7], [8], [9]]. This leaves open the debate as whether or not to adopt a multi-segmental approach for the lumbar spine.

Moreover, current analyses have mostly focused on the lumbar spine in isolation, as the site of pain, overlooking other spine regions. Crosbie et al. [10] showed that upper and lower thoracic spine kinematics amplitudes are significantly reduced in patients with LBP during downward reaching whilst no differences were observed in the lumbar region. Similarly, other studies found regional thoracic kinematics differences between LBP and controls during sit-to-stand (STS) [4] and range of motion (ROM) tasks [11,12]. Similar findings were also noted when the thoracic spine was considered as a single segment [13,14]. These results demonstrate the importance of assessing thoracic spine motion alongside the lumbar spine in LBP groups in order to enhance understanding of movement coupling.

However, when assessing the whole spine using a multi-segmental approach, consideration should be given to the practicality of the approach, including errors and time implications of attaching markers to track different spine regions, as well as, the technical limitations of motion capture systems that may constrain the measurement resolution at the intervertebral level.

Therefore, it is important to establish the necessity of a multi-segmental approach to determine at which level of detail the analysis should be conducted. A study of ROM tasks in healthy volunteers found that clusters of markers at C7, T6, T12 and L5 are sufficient to characterise thoracic spine kinematics [15]. Similarly a multi-segmental lumbar model is required when analysing gait and a prone hip extension exercise as it was shown that the upper and lower lumbar segments move distinctively during these tasks [16].Varying levels of correlations between upper and lower lumbar segments depending on the task have been reported [16], showing task-dependency in segmental redundancy (i.e.: the movement pattern of adjacent segments is similar so as to be considered redundant and therefore one segment could be eliminated from the analysis). Moreover, patients with LBP have different movement patterns to controls depending on the tasks analysed [3]. The population assessed may also therefore influence segmental redundancy. Consequently, it is relevant to explore segmental redundancy in the combined thoracic and lumbar spine segments during functional tasks and to evaluate if the same results are obtained in a patient population. A better understanding of the requirement for multi-segmental analysis of the spine could guide planning of future studies and avoid missing clinically-relevant information.

The aim of this study was to assess the correlation between the movement of the upper and lower thoracic and lumbar spine segments in order to evaluate segmental redundancy between adjacent segments in both healthy and participants with LBP for a series of different tasks.

## 2 Methods

### 2.1 Participants

Forty volunteers were recruited: 20 healthy controls (age:28 ± 7.6 years, body mass:66.2 ± 12 kg, height:1.72 ± 0.11 m, 10 female) and 20 non-specific chronic patients with LBP (age:41 ± 10.7 years, body mass: 74.1 ± 19.5 kg, height:1.68 ± 10.7 m, 4 female). Non-specific LBP was defined as pain in the lower back region for which it was not possible to identify a specific cause (e.g. prolapsed disc, sciatica, tumour, spinal stenosis). Participants were excluded if they had neurological diseases, severe musculoskeletal deformities in the lower limbs or spine, spinal fractures, and back surgery. Ethics approval was obtained from the North-West Preston Research Ethics Committee. All participants provided written informed consent prior to participation.

### 2.2 Experimental procedures

**Fig. 1:**
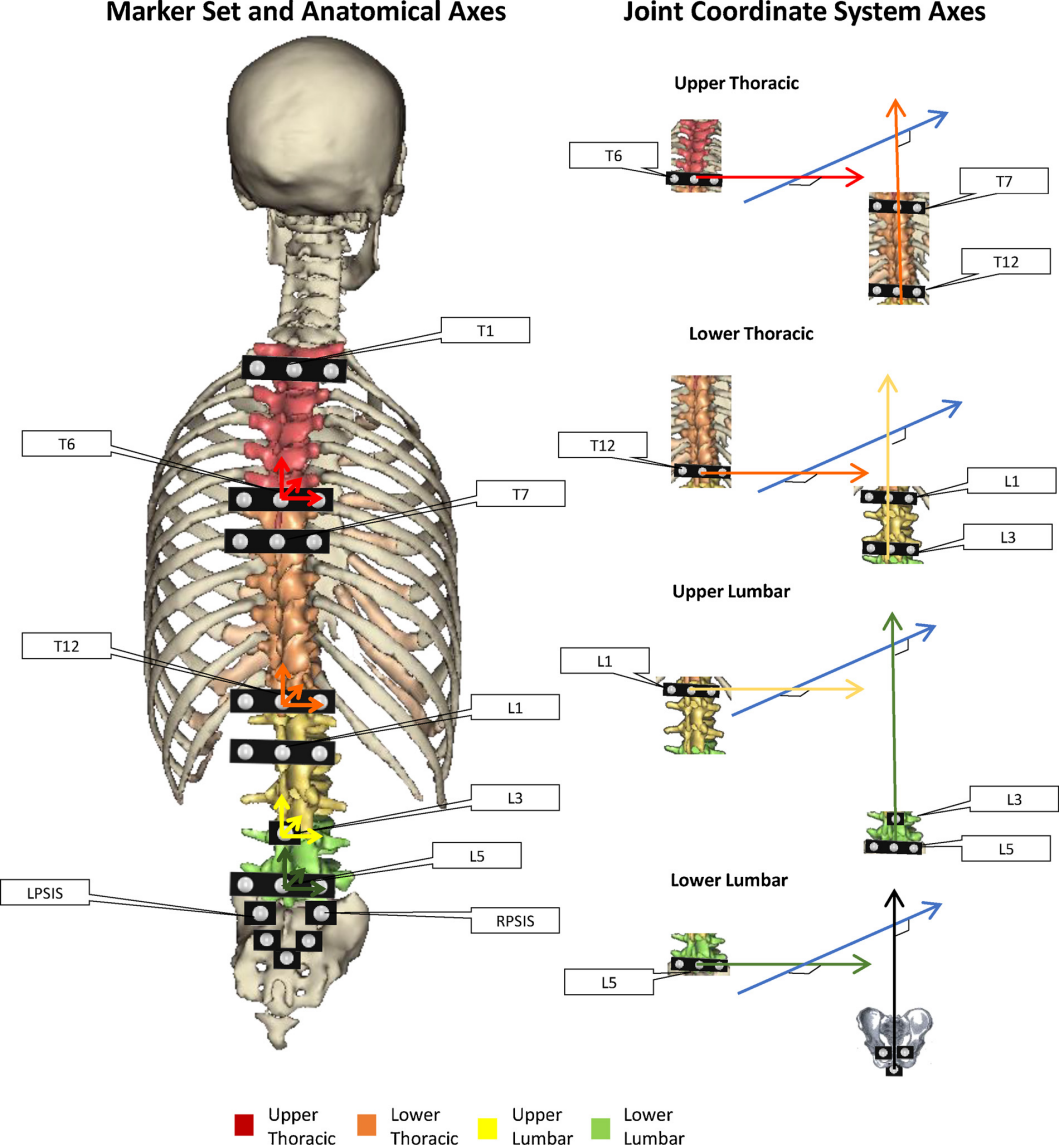
Schematic of marker placement and spine anatomical frames of reference (left side); Joint coordinate system axes of rotation for each spine segment considered (right side).

Spine Kinematic data were collected with a 10 camera 3-D motion capture system (Vicon, Oxford Metrics, Oxford, UK) operating at 100 Hz. Spherical retro-reflective markers (diameter:14 mm) were positioned over the spine on the spinous processes of T1, T6, T7, T12, L1, L3, L5 (Fig. 1). Markers were attached on plastic strips to form a triad with the central marker at 2.5 cm distance from each of the lateral markers. The central marker was always positioned on the spinous processes which were identified following palpation guidelines [17]. Additionally, the pelvis movement was tracked using a three-marker rigid cluster placed over the sacrum (Fig. 1); markers on the left and right anterior and posterior iliac spine (ASIS; PSIS) were referenced to this cluster during static calibration and then removed. Data collection started with a calibration trial during quiet upright standing. Participants then performed three trials each of: walking at a self-selected pace, STS, and lifting a 5 kg box. These tasks were chosen, because they represent some of the tasks frequently described as painful by people affected by LBP; they showed an increased ability to discriminate between LBP and healthy groups; and because they are some of the activities most often repeated throughout a day [[2], [3], [4], [5], [6]]. Standardised instructions were used each time and practice was offered for each task. Participants with LBP also completed the Oswestry Disability Index (ODI) to quantify disability resulting from their back pain [18].

### 2.3 Data processing and analysis

Markers trajectories were reconstructed, and gap filled using Nexus software (Vicon, Oxford Metrics, Oxford, UK). A Woltring’s general cross-validatory quintic smoothing spline with a predicted mean-squared error of 15 mm was used to filter marker trajectories [19]. Upper and lower thoracic and lumbar spine kinematics were calculated using the following spine model (Fig. 1): the upper thoracic segment (UT), between T1 and T6, was defined with its origin at T6, a vertical axis from T6 to T1, a horizontal axis passing through the markers to the left and right of T6 and an anterior/posterior axis mutually perpendicular to the other two axes; the lower thoracic segment (LT, T7-T12) has its origin at T12, a vertical axis from T12 to T7, a horizontal axis passing through the markers at the left and right of T12 and an anterior/posterior axis mutually perpendicular to the other two axes; the upper lumbar segment (UL, L1-L3) was defined with its origin at L3, a vertical axis from L3 to L1, a horizontal axis passing through the markers to the left and right of L1 and an anterior/posterior axis mutually perpendicular to the other two axes; the lower lumbar segment has its origin at L5, a vertical axis from L5 to L3, a horizontal axis passing through the markers to the left and right of L5 and an anterior/posterior axis mutually perpendicular to the other two axes. The pelvis segment was defined as per recommendations: the origin was the midpoint between the ASISs, horizontal axis passing through the left and right ASISs, antero/posterior axis passing through the midpoints of the two ASISs and PSISs and perpendicular to the horizontal axis, vertical axis mutually perpendicular to the other two [20]. The joint coordinate system [22] was used to calculate 3D joint angles (Fig. 1b). The upper thoracic and upper lumbar angles were defined as the relative movement between the upper thoracic and upper lumbar segment with respect to the lower thoracic and lower lumbar segment respectively. The lower thoracic angles were defined based on the position of the lower thoracic segment relatively to the upper lumbar segment and the lower lumbar angles as the angles between the lower lumbar segment and the pelvis segment. This spine model was previously tested for intra- and inter-rater reliability with intra-class correlation coefficients over 0.6 [21].

**Fig. 2:**
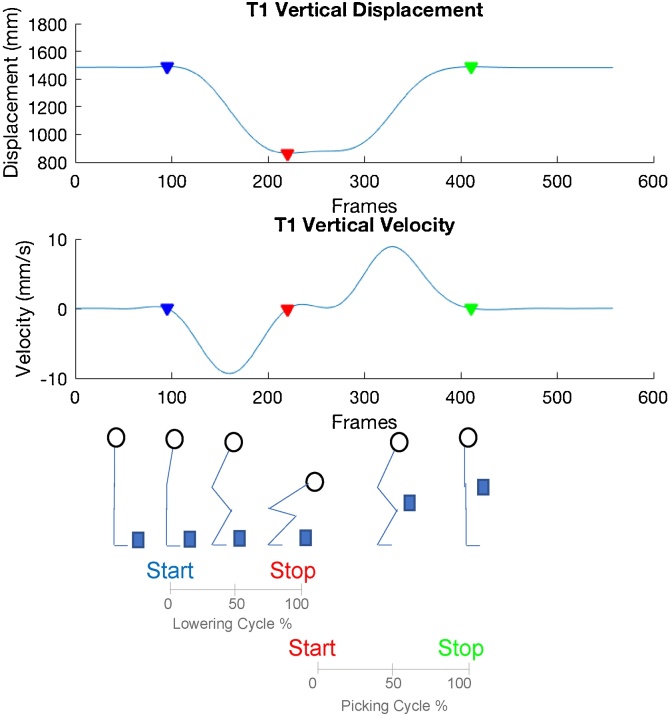
Detection of picking and lowering phase cycles based on T1 vertical displacement and velocity. Coloured triangles show the beginning and end of each phase.

**Fig. 3:**
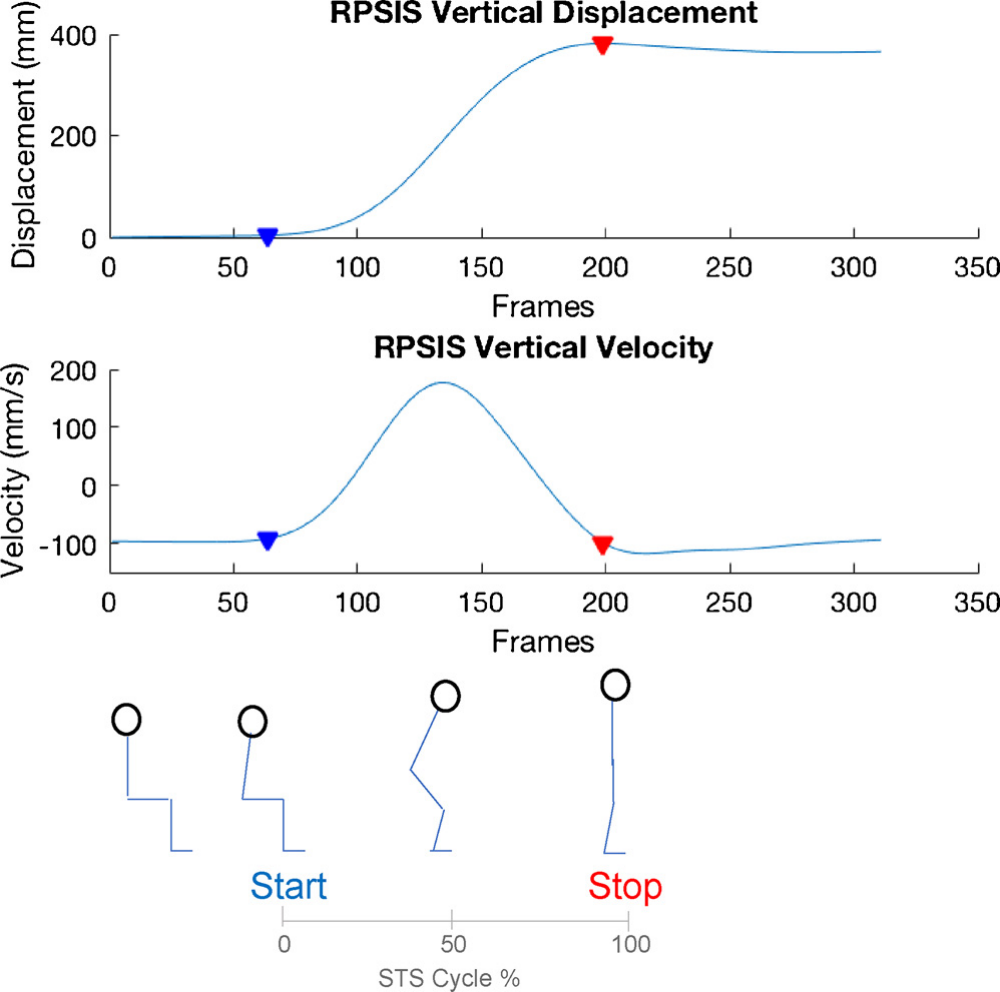
Detection of STS cycle based on the right PSIS vertical displacement and velocity. Coloured triangles show the beginning (blue) and end (red) of STS phase.

Data were time normalised to the duration of each task to 100 data points. For the walking trials, heel strike events to define left and right gait cycles were determined using the horizontal heel displacement method [23]. Left and right walking kinematics were averaged as similar patterns were observed. The lifting task was split into lowering and picking phases (Fig. 2). The beginning and end of these and STS were determined with a custom code using a combination of markers (PSIS, T1), displacement peak and troughs and changes in velocity to identify each cycle duration (Fig. 2, Fig. 3).

Cross-correlation analyses were conducted on the time histories of 3D angle data between pairings of adjacent spine segments to assess segmental redundancy. A total of 3 analyses were performed (UT vs LT; LT vs UL; UL vs LL). Cross-correlation analysis determines the spatial and/or temporal similarity between two signals [24] and here it is used to assess the extent of the association between kinematics time series of two adjacent segments. Cross-correlation coefficients (R_xy_) at time lag zero, when the time series are aligned, were extrapolated to quantify the strength of their relationship [15] for all tasks performed. The mean R_xy_ over 3 task repetitions for each participant were calculated and then averaged across healthy and LBP groups to enable a comparison between segmental redundancy for those with or without a musculoskeletal condition that may affect spine movement. Calculations were performed with Matlab (The MathWorks, Inc., Natick, USA).

The ranges of motion (ROMs) in all 3 planes for each spine segment were calculated as the difference between the maximum and minimum angles. The ROM means were determined across the 3 trials of each task and in each segment and used to calculate correlation coefficients between adjacent segments. Spearman's rank correlation and Pearson product moment correlation coefficients (R_ROM_) were calculated for non- and normally distributed data respectively in SPSS (IBM, Armonk, USA). The normality of the data was assessed using Q—Q plots and the Shapiro-Wilk test. Independent t-tests were performed to test for differences in groups demographics. A significance level of 0.05 was used for the analyses. Correlation coefficients (R_xy_, R_ROM_) were interpreted as follows [16]: very strong (0.80–1.00), strong (0.60–0.79), moderate (0.40–0.59), weak (0.20–0.39), and very weak (0.00–0.19).

## 3 Results

There were no significant differences between groups in height (p-value = 0.29) and body mass (p = 0.20), but the LBP group was 13 years older than the controls (p < 0.05). Five participants with LBP reported moderate disability based on the ODI score system (21%≤ODI≤40%) and 15 minimal disability with ODI score ≤20%.

Table 1 shows R_xy_ values for the three anatomical planes of rotation for both participant groups during the tasks performed. Different behaviours were observed across segment pairings, tasks and participant groups.

**Table 1:** R_xy_ mean (SD) values between spine adjacent segments for each task in both groups assessed in the three anatomical planes. Bold values represent strong to very strong correlations.

	R_xy_																	
	Frontal Plane						Transverse Plane						Sagittal Plane					
	UT/LT		LT/UL		UL/LL		UT/LT		LT/UL		UL/LL		UT/LT		LT/UL		UL/LL	
	H	LBP	H	LBP	H	LBP	H	LBP	H	LBP	H	LBP	H	LBP	H	LBP	H	LBP
**Walk**	0.53 (0.36)	0.25 (0.53)	0.25 (0.54)	0.22 (0.38)	0.40 (0.31)	0.27 (0.48)	0.14 (0.38)	0.34 (0.40)	**0.87** **(0.10)**	**0.78** **(0.37)**	0.08 (0.55)	0.02 (0.45)	−0.08 (0.31)	−0.06 (0.38)	0.01 (0.38)	0.01 (0.47)	−0.25 (0.34)	−0.27 (0.31)
**STS**	−0.14 (0.44)	−0.31 (0.43)	0.31 (0.51)	0.13 (0.50)	−0.10 (0.52)	−0.17 (0.50)	−0.34 (0.36)	−0.42 (0.41)	**0.60** **(0.29)**	**0.60** **(0.30)**	−0.18 (0.36)	−0.07 (0.53)	0.23 (0.38)	0.41 (0.42)	**0.89** **(0.12)**	**0.61** **(0.51)**	0.25 (0.50)	0.02 (0.45)
**Picking**	−0.16 (0.50)	−0.21 (0.53)	0.26 (0.58)	0.23 (0.47)	−0.24 (0.52)	−0.14 (0.50)	−0.28 (0.50)	−0.10 (0.50)	0.50 (0.50)	0.52 (0.53)	−0.13 (0.56)	−0.06 (0.50)	−0.47 (0.56)	−0.19 (0.40)	**0.94** **(0.17)**	**0.81** **(0.44)**	**0.65** **(0.45)**	0.25 (0.55)
**Lowering**	−0.24 (0.52)	−0.25 (0.43)	0.27 (0.48)	0.19 (0.45)	−0.18 (0.54)	−0.21 (0.44)	−0.46 (0.40)	−0.15 (0.55)	0.44 (0.47)	0.49 (0.43)	−0.12 (0.52)	−0.19 (0.47)	−0.01 (0.52)	0.34 (0.56)	**0.90** **(0.27)**	**0.80** **(0.45)**	**0.64** **(0.56)**	0.36 (0.29)

H: Healthy; LBP: Low Back Pain; UT:Upper Thoracic; LT: Lower Thoracic, UL: Upper Lumbar; LL: Lower Lumbar.

*Frontal plane*: Very weak to weak correlations were observed for all three segment pairings across all tasks in the LBP group and during STS and lifting phases only in the controls. During walking the controls displayed moderate correlations in UT/LT and UL/LL pairings and weak correlation between the LT and UL segments.

*Transverse plane*: The LT/UL pairings showed strong to very strong correlations during walking and STS in both groups and moderate for lowering and picking phase. In all other cases very weak to weak correlations were observed apart from a moderate negative correlation in the UT/LT pairing in the healthy during lowering and in the LBP group during STS.

*Sagittal plane*: During walking all segment pairings moved in a very weak to weak correlated fashion across the two groups. Strong to very strong correlations were observed for STS, lowering and picking for LT/UL pairing in both groups. Controls also displayed strong correlation during picking and lowering in the UL/LL pairing which showed instead weak correlation in participants with LBP.

**Fig. 4:**
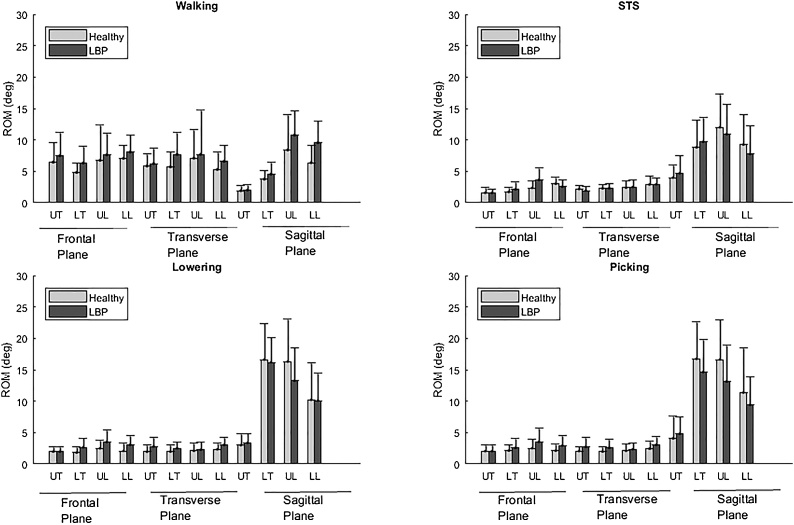
ROM mean (±standard deviation) of thoracic and lumbar spine segments in the 3 anatomical planes for all tasks analysed for people with (grey bars) and without LBP (light grey bars).

3D ROM mean values of all analysed segments are reported in Fig. 4 for both groups. Correlation coefficients, R_ROM_, between segment pairings showed a similar variability to R_xy_ across segment couplings, tasks and participants groups (Table 2). Strong to very strong correlations were observed only in 2 cases out of 72 analysed, these are highlighted in Table 2 with bold font. Most R_ROM_ values (55/72) demonstrated weak to very weak correlations.

**Table 2:** R_ROM_ values between spine adjacent segments for each task in both groups assessed in the three anatomical planes. Bold values represent strong to very strong correlations. Significant correlations are indicated with *.

	R_ROM_																	
	Frontal Plane						Transverse Plane						Sagittal Plane					
	UT/LT		LT/UL		UL/LL		UT/LT		LT/UL		UL/LL		UT/LT		LT/UL		UL/LL	
	H	LBP	H	LBP	H	LBP	H	LBP	H	LBP	H	LBP	H	LBP	H	LBP	H	LBP
**Walk**	−0.01	0.04	0.30	0.32	0.03	0.06	0.21	0.42	0.80*	0.34	0.21	−0.20	0.30	0.25	0.43	0.36	0.58*	0.40
**STS**	0.01	0.50*	0.13	−0.12	0.34	0.15	0.36	0.46*	0.38	0.33	−0.30	0.22	0.19	0.02	0.34	0.26	−0.24	0.20
**Picking**	0.44	−0.25	0.39	0.32	0.49*	−0.04	0.13	0.12	0.51*	0.51*	0.30	0.30	0.42	0.37	0.36	0.37	−0.23	0.13
**Lowering**	0.20	0.08	0.30	0.10	0.06	−0.32	0.13	0.23	0.47*	0.64*	0.15	−0.11	0.40	0.44	0.20	0.22	−0.21	0.21

UT:Upper Thoracic; LT: Lower Thoracic, UL: Upper Lumbar; LL: Lower Lumbar.

## 4 Discussion

The assessment of spinal movement can enhance our understanding of spine pathologies, such as LBP. Previous studies analysing the lumbar spine have considered it as a single rigid segment [3]. However, more recent LBP research has indicated regional differences in lumbar spinal movement and has proposed assessing a more detailed regional motion of the spine [2,[4], [5], [6],[10], [11], [12], [13], [14]]. Such models are complex therefore it is important to establish what level of complexity is required from a multi-segmental model of the spine and more specifically if this is influenced by task and the presence and absence of low back pain. This study investigated the need for a spine multi-segmental model using cross-correlation and correlation analyses of spine kinematic time series and ROM between spine adjacent segments in healthy and participants with LBP.

The findings showed that different spine regions move in an uncorrelated fashion thereby demonstrating that the use of a single rigid segment for both the thoracic and lumbar spine is not representative and as such is unrealistic. This agrees with previous studies that looked at segmental redundancy within the thoracic spine during ROM manoeuvres [15] and within the lumbar spine during gait and prone hip extension exercise in healthy [16]. In addition to previous work, the current study looked at different functional tasks showing task dependency in spinal segments’ movement and extended the analysis to a pathological population. Our analysis of three of the most commonly performed daily tasks permits these findings to be incorporated in future functional motion analysis studies. A limitation of our work is that our LBP group was composed of individuals with only moderate to low disability. However, differences between the two groups, anatomical planes of motion and tasks analysed were observed and one can surmise that such difference would be greater in patients with greater disability [3]. For instance, whereas UL/LL pairings always showed weak to very weak correlation in LBP participants the same cannot be said for controls.

To enhance the ability of biomechanical studies to identify movement pattern differences between LBP and healthy groups, the adoption of a multi-segmental analysis of the spine is advisable. This is in accordance with previously conducted movement studies which found statistically significant differences in ROM between LBP and controls when the lumbar segment was analysed as two regions [2,[4], [5], [6]] but not when considered as one single rigid segment [11,25,26]. Therefore, since we cannot *a priori* confirm where the redundancy occurs, it is not appropriate to assume segmental redundancy when assessing a pathological population.

One could argue that the spine could be divided even further and consider each vertebra separately as an individual segment. However, the technical limitations of using a motion capture system which is unable to capture markers that are too close to each other without significant error, and the impracticability of the time required to position markers in study participants means that this is currently not possible. Moreover, higher number of markers increases the likelihood of marker misplacement and hence errors in the outcomes. Schinkel-Ivy et al. [15] used clusters/markers at C7, T3, T6, T9, T12, and L5 to show that a set of clusters at C7, T6, T12, and L6 would be sufficient to describe the thoracic movement. We considered both the thoracic and lumbar spine which were divided based on specific spinal processes (T1, T6, T7, T12, L1, L3, L5) similar to the one listed by Schinkel-Ivy et al. [15]. Those spinal processes were selected as they represent easily identifiable anatomical landmarks along the spine to ensure repeatability across measurements, to minimise, where possible, spinal processes that are shared between adjacent spine segments and to be compliant with previously used spine models [2,[4], [5], [6],^8^,^10^,^11^,^15^]. Finally, the ROM values found in this study agree with prior published values [2,4,10,13,26]. Discrepancies with previous results, where observed, can be explained by different protocol procedures, variation in biomechanical models used, and differences in data processing procedures as well as population assessed. Nevertheless, the model adopted permits the assessment of regional movement within the thoracic and lumbar spine as well as their relationships.

There are limitations in this study that need to be considered. Spinal kinematics may have been affected by errors due to soft tissue artefact derived from skin marker movements with respect to the underlying spinal processes, and this could have affected the different regions differently. Although we could not quantify the artefact directly in this study and its effect on spinal kinematics, previous studies estimated errors around 10 mm for spine markers and showed consistent and correlated results between skin markers and imaging gold-standard systems [27,28]. Participants from both groups were likewise exposed to instrumentation and soft tissue artefacts therefore it is not expected that the differences found between groups are due those errors. Nevertheless, experimental errors could have occurred and concealed further differences between the two groups. Although ideally each vertebra should be considered separately in the analysis of the spine, we only considered four segments. This was a compromise between a multi-segmental approach and applicability of this method in clinical practice with technical constraints. Further investigations are required to prove the need of a more detailed model. The findings showed task-dependency of segmental redundancy. In this study, we considered three common daily tasks, but attention should be paid if results are to be translated to tasks not assessed in this study. Participants with LBP showed low to moderate disabilities and therefore differences may be enhanced in a more heterogeneous group. Finally, groups differed in age and this could have also affected their kinematics; age matching should be considered in future studies. However, no differences in the gait, lifting and STS speeds were found between the groups (*p-values* range: 0.13-0.74). Our cross-correlation and correlation coefficient interpretation was based on one arbitrarily selected classification criterion used in a similar study [16], the use of other criteria may lead to a different interpretation of the results.

## 5 Conclusions

This study shows that spine segmental redundancy should not be assumed and a multi-segmental analysis of the spine may be required if movement characteristics are to be fully understood. Segmental redundancy depends on task, anatomical planes and on the population group assessed as the same conclusions could not be drawn for healthy and participants with LBP. Therefore, segmental redundancy cannot be accepted when assessing pathologies, as due to different movement couplings it cannot be confirmed *a priori* where the redundancy occurs.

## Conflict of interest statement

Nothing to declare.
